# Potential pharmacogenomic targets in bipolar disorder: considerations for current testing and the development of decision support tools to individualize treatment selection

**DOI:** 10.1186/s40345-020-00184-3

**Published:** 2020-07-04

**Authors:** Alfredo B. Cuéllar-Barboza, Susan L. McElroy, Marin Veldic, Balwinder Singh, Simon Kung, Francisco Romo-Nava, Nicolas A. Nunez, Alejandra Cabello-Arreola, Brandon J. Coombes, Miguel Prieto, Hannah K. Betcher, Katherine M. Moore, Stacey J. Winham, Joanna M. Biernacka, Mark A. Frye

**Affiliations:** 1 Department of Psychiatry, University Hospital, Universidad Autonoma de Nuevo Leon, Monterrey, Mexico; 2 Department of Psychiatry and Psychology, Mayo Clinic, 200 First Street SW, Rochester, MN 55905 USA; 3 Lindner Center of HOPE and Department of Psychiatry, University of Cincinnati, Cincinnati, OH USA; 4 Department of Health Sciences Research, Mayo Clinic, Rochester, USA; 5 Department of Psychiatry, Universidad de los Andes, Santiago, Chile

**Keywords:** Pharmacogenomic testing, Bipolar disorder, Pharmacogenetics

## Abstract

**Background:**

Treatment in bipolar disorder (BD) is commonly applied as a multimodal therapy based on decision algorithms that lack an integrative understanding of molecular mechanisms or a biomarker associated clinical outcome measure. Pharmacogenetics/genomics study the individual genetic variation associated with drug response. This selective review of pharmacogenomics and pharmacogenomic testing (PGT) in BD will focus on candidate genes and genome wide association studies of pharmacokinetic drug metabolism and pharmacodynamic drug response/adverse event, and the potential role of decision support tools that incorporate multiple genotype/phenotype drug recommendations.

**Main body:**

We searched PubMed from January 2013 to May 2019, to identify studies reporting on BD and pharmacogenetics, pharmacogenomics and PGT. Studies were selected considering their contribution to the field. We summarize our findings in: targeted candidate genes of pharmacokinetic and pharmacodynamic pathways, genome-wide association studies and, PGT platforms, related to BD treatment. This field has grown from studies of metabolizing enzymes (i.e., pharmacokinetics) and drug transporters (i.e., pharmacodynamics), to untargeted investigations across the entire genome with the potential to merge genomic data with additional biological information.

**Conclusions:**

The complexity of BD genetics and, the heterogeneity in BD drug-related phenotypes, are important considerations for the design and interpretation of BD PGT. The clinical applicability of PGT in psychiatry is in its infancy and is far from reaching the robust impact it has in other medical disciplines. Nonetheless, promising findings are discovered with increasing frequency with remarkable relevance in neuroscience, pharmacology and biology.

## Background

Bipolar Disorder (BD) is a complex chronic mood disorder where patient’s lives are variably associated with episodic recurrence (Angst and Sellaro 2000; Judd and Akiskal 2003), psychosocial and functional disability (Tohen et al. 2000; Zarate et al. 2000), and substantial morbidity and mortality especially in the depressive phase of illness (Frye 2011). While there have been advances in disease classification of illness subtype (i.e., BD-I, BD-II), symptom specifier (i.e., anxious distress, mixed features, rapid cycling, peripartum onset), and increasing recognition of high rates of comorbidity, the illness remains highly heterogeneous within patient groups and within a single patient’s longitudinal course of illness (Malhi et al. 2018). Moreover, the Diagnostic and Statistical Manual of Mental Disorders (DSM-5) classification is not defined by an underlying pathophysiology and as such, limits the current understanding of neurobiological mechanisms of differential illness presentation and development of biomarkers of disease burden (Frey et al. 2013; Harrison et al. 2018).

There is no greater psychotropic pharmacopoeia in psychiatry than that of BD. Treatment selection with lithium, mood stabilizing anticonvulsants, mood stabilizing atypical antipsychotics, typical antipsychotics, unimodal antidepressants, and benzodiazepines, most commonly as a multimodal therapy, will be based on a number of factors including: clinical evidence base, phase of illness and symptom severity, BD-I vs BD-II subtype, level of cyclicity, and additional mental health and medical diagnoses that may impact efficacy and/or side effect burden. Molecular drug mechanisms of action, biomarkers of treatment response or adverse events, are not part of any clinical decision algorithm in BD. Oftentimes, polypharmacotherapy is necessary to achieve remission (Frye et al. 2000). However, multimodal drug therapy is challenging given the potential for pharmacokinetic drug–drug interactions and cumulative side effect burden. Developing biomarkers to individualize treatment in hopes of increasing rates of remission, tolerability, and adherence or mitigate serious drug related adverse event risk would represent a paradigm shift in current clinical practice models for both prescribers and patients.

Pharmacogenetics/genomics study the individual genetic variation associated with drug response. This field has grown from studies of metabolizing enzymes (i.e., pharmacokinetics) and drug transporters (i.e., pharmacodynamics), to untargeted investigations across the entire genome with the potential to merge genomic data with additional biological information (Weinshilboum and Wang 2017). Additional focus on a patient’s individual biology, vs solely broad Food and Drug Administration (FDA) indication labeling, may facilitate patient care with the “right drug, right dose, right time” (Bielinski et al. 2014). There are early pharmacogenomic studies in most treatment classes in BD (Pisanu et al. 2018b). This is of special importance in BD, where early intervention can have a positive impact in the progression of the disease (Post 2018).

For this selective review of pharmacogenomics in BD we searched PubMed from January 2013 to May 2019, to identify studies reporting on BD and pharmacogenetics, pharmacogenomics and pharmacogenomic testing (PGT). The authors selected studies based on their contribution to the field. We focus on candidate genes and genome wide association studies (GWAS) of pharmacokinetic drug metabolism and pharmacodynamic drug response/adverse event, and the potential role of decision support tools (DST) that incorporate multiple genotype/phenotype drug recommendations. The Clinical Pharmacogenomics Implementation Consortium (CPIC) (Relling and Klein 2011), was established as a joint effort between PharmGKB and the Pharmacogenomics Research Network (PGRN) to develop peer-reviewed guidelines for implementation of PGT, will be reviewed to illustrate early examples of clinical practice recommendations.

## Targeted candidate genes in pharmacokinetic drug metabolism

The Cytochrome P450 (CYP) superfamily of proteins (2D6, 2C9, 2C19, 3A4) is one of the most important enzymatic classes responsible for phase I drug-metabolism, and thus, relevant to most of psychiatric medication’s metabolism and bioactivation (Spina and de Leon 2015). The notable exception, given its lack of metabolism is lithium. Early efforts in pharmacogenetics were, in part, dedicated to CYP genotyping/metabolic phenotyping with the goal of operationally defining phenotype classes that would be associated with response and side effects. These CYP phenotypic classifications [i.e., poor, intermediate, extensive (normal), and ultra-rapid] are the most common application of commercial PGT in psychiatry (Eum et al. 2016), however, no standardized model prevails to date (Gaedigk et al. 2017). It is important to acknowledge that race and ethnicity are important sources of variability between populations in allele composition and frequency of CYP genes. Inter-individual variability is even larger than that observed between ancestries producing large ranges of CYP activity among individuals. Moreover, CYP genes are strongly influenced by complex environmental factors that cannot be accounted by current PGT (McGraw et al. 2018). Nonetheless, the poor metabolizer phenotype and use of antidepressants has been the focus of a number of forensic cases of possible drug related fatality (i.e., 2D6), has driven a number of FDA drug label revisions (i.e., 2D6, 2C19) related to arrhythmia risk, and has been speculated, given black box warning for antidepressants in young adults, as a possible mechanism of treatment emergent suicidal ideation and antidepressant induced mania (AIM), both phenomena often identified early in the course of treatment [reviewed by (Nassan et al. 2016)]. Table 1 summarizes the evidence on pharmacokinetic PGT by drug class. Comprehensive sources on CYP PGT in psychiatry (Spina and de Leon 2015; Eum et al. 2016; Solomon et al. 2019) and *CYP2D6* genetic variation considerations (Nofziger et al. 2020) are reviewed elsewhere.

**Table 1:** Potential content of commercial PGT+

Gene*	Drug-class	Use in bipolar disorder	Trial on BD population	CPIC level of evidence	FDA label
CYP2D6	Second-Generation Antipsychotics (SGAs)	Positive association of CYP2D6 genotype and tardive dyskinesia (Fleeman et al. 2011)	No	Not included	No
	SGAs	Positive association of CYP2D6 genotype and weight gain (Fleeman et al. 2011)	No	Not included	No
	Aripiprazole	Changes in serum concentrations in poor metabolizers (Hendset et al. 2007)	No	B	Actionable
	Risperidone	Changes in serum concentrations in poor metabolizers (Eum et al. 2016)	No	B	Informative
	Tricyclic Antidepressants	Association of changes in serum concentrations with metabolizer phenotype (Hicks et al. 2017)	No	A, Guideline	Informative
	Selective Serotonin Reuptake Inhibitors (SSRIs)	Association of changes in serum concentrations with metabolizer phenotype (Hicks et al. 2015)	No	C-D, Guideline	Informative
		Antidepressant induced mania (AIM) in 3 poor metabolizer bipolar patients (Sanchez-Iglesias et al. 2016)	Yes (mixed)	Not included	No
CYP2C19	Tricyclic Antidepressants	Association of changes in serum concentrations with metabolizer phenotype (Hicks et al. 2017)	No	A, Guideline	No
	Citalopram, Escitalopram	Association of changes in serum concentrations with metabolizer phenotype (Hicks et al. 2015)	No	A, Guideline	Actionable
	Escitalopram	A retrospective study of 2087 gentoyped patients showed that poor and ultrarapid CYP2C19 metabolizers seem to predict greater switching from escitalopram to another agent (Jukic et al. 2018)	Multiple Diagnosis	Not included	No
	Sertraline	Association of changes in serum concentrations with metabolizer phenotype (Hicks et al. 2015)	No	B, Guideline	No
HLA-B	Carbamazepine	There is a strong recommendation by the CPIC of not to use carbamazepine in carbamazepine naive patients, with HLA-B*15:02 positive subjects given a “Greater risk of carbamazepine-induced SJS/TEN”. Proceed with caution in HLA-B*15:02 negative subjects, depending on HLA-A*31:01 genotype; there may be an average risk in negative vs. Higher risk in positive alleles (Phillips et al. 2018) HLA-B variants have been associated with carbamazepine induced agranulocytosis/granulocytopenia in European populations (Goldstein et al. 2014)	No	A	Actionable
CYP2C9	Valproate	The loss-of-function alleles, CYP2C9*2 or CYP2C9*3, display significant reduction in valproate metabolism in children; furthermore, low CYP2C9 expression in patients with CYP2C9*1/*1 genotype also leads to a decrease in valproate metabolizing capacity (Monostory et al. 2019)	No	No	No
GRIK4	Citalopram	Initial modest association observed in the STAR*D trial (Paddock et al. 2007). Meta-analysis results showed that the C allele appeared more frequently than the T allele in responders to treatment (OR: 1.22; 95% CI 1.035–1.445; z = 2.36; p = 0.018) (Kawaguchi and Glatt 2014)	No	Level D	No
	Haloperidol	Early and modest evidence of association with antimanic effect of haloperidol in BD (Drago et al. 2013)	Yes	No	No
DRD2	Aripiprazole, Risperidone	C/C homozygotes improved in positive symptoms more than the T carriers during 12 weeks of treatment with aripiprazole or risperidone, C/C homozygotes developed more akathisia during treatment with aripiprazole, rolactin elevation in males treated with risperidone, in that C/C homozygotes had lower elevation of prolactin compared to the T carriers (Zhang et al. 2015)	First-episode psychosis	C (Risperidone)	No
	Antipsychotics	A meta-analysis of 698 schizophrenia patients, found that Del allele carrier of the -141C Ins/Del polymorphism, were significantly associated with poorer antipsychotic drug response, compared to the Ins/Ins genotype, OR = .65, p = 0.03 (Zhang et al. 2010)	No	C (Risperidone)	
SLC6A4	Antidepressants	A meta-analysis of 1034 bipolar patients and antidepressant remission rates reported reduced anti-depressive remission rates in S-carriers of the serotonin transporter promoter polymorphism (OR = 0.64, p = .006, I2 = 0.0%) (Rao et al. 2019)	Yes	Not included	No
		A 6-study (453 bipolar patients) meta-analysis demonstrated a marginally significant evidence of association of the S allele with AIM (OR = 1.35; 95% CI 0.99–1.85; P = 0.059) (Frye et al. 2015)	Yes	Not included	No
EPHX1	Carbamazepine	Carriers of the SCN1A IVS5-91GA variant or of EPHX1 c.337TC variant presented significantly lower levels of plasma CBZ compared to carriers of the common alleles (0.71 ± 0.28 vs 1.11 ± 0.69 μgmL per mgKg for SCN1A IVS5-91 AA vs GG and 0.76 ± 0.16 vs 0.94 ± 0.49 μgmL per mgKg for EPHX1 c.337 CC vs TT; P0.05 for both) (Daci et al. 2015)	No	D	No
HLA-A	Carbamazepine	Due to “Greater risk of carbamazepine-induced SJS/TEN” by CPIC, proceed with caution in HLA-B*15:02 negative subjects, depending on HLA-A*31:01 genotype; there may be an average risk in negative vs. Higher risk in positive alleles (Phillips et al. 2018)	No	A	Actionable
HTR2A	Antidepressants	A meta-analysis found association of greater antidepressant response in major depressive disorder, for the dominant models of rs6313 5HTR2A-T > C polymorphism (OR = 1.62; 95% CI 1.21–2.18; P = 0.008) and rs7997012G > A (OR = 1.92; 95% CI 1.02–3.61; P = 0.044) (Lin et al. 2014)	No	D	No
HTR2C	Clozapine, Olanzapine, Risperidone	A meta-analysis found significant association betwen the C allele of the HTR2C rs1414334:C > G polymorphism (OR = 2.44; 95%CI [1.48, 4.00]; P = 0.0004; I2 = 0), the HTR2C -697 G/C polymorphism (OR = 1.54; 95%CI [0.99, 2.40]; P = 0.05; I2 = 0), and olanzapine/clozapine/risperidone-induced metabolic syndrome (Ma et al. 2014)	No	D	No
OPRM1	Naltrexone	Several trials have found an association between the A118G rs1799971 polymorphism and naltrexone response (Patriquin et al. 2015), review	No	C/D	No
ABCB1	Antidepressants	Multiple genetic variants have been explored. Mixed evidence of association with less dose for remission, response, time to remission and remission in treatment of unipolar depression with antidepressants. The majority of evidence found associations with side effects and tolerability. (For a comprehensive review see (Bruckl and Uhr 2016)	No	A/B	No
COMT	SSRIs	Significant association between rs13306278 and remission (P = 0.038) in 1914 depressed patients from STAR*D genotyped for COMT (Ji et al. 2012)	No	C	No
CYP3A5	Alprazolam	In a study of 19 healthy volunteers, CYP3A5 non-expressors had a lower alprazolam clearance compared carriers of the CYP3A5*1/*1 and CYP3A5*1/*3 alleles (Park et al. 2006)	No	C	No
UGT1A4	Lamotrigine	A meta-analysis found no associations between concentration to dose ratio (CDR) values and different polymorphisms of UGT1A4. The non-pediatric population showed a non-significant trend of association between UGT1A4 142T > G WT and higher CDR (Kim and Kim 2019)	No	D	No

*The genetic variants genotyped in PGT for each gene are many times unknown, thus interpretation must be done with caution

**These recommendations follow the CPIC Guidelines

### CYP2D6

*CYP2D6* is the most involved CYP isoform in psychiatric drug metabolism, and hundreds of *CYP2D6* polymorphisms and copy number variations have been identified, many of which have unknown effects (McGraw et al. 2018). Multiple antidepressants and antipsychotics of different classes are its substrates, and CYP2D6 is inhibited strongly by fluoxetine, paroxetine, perphenazine and thioridazine. A recent study by Gaedgik et al. (2017) analyzed 177 reports of a world-wide population compromised of approximately 60,000 unrelated subjects and categorized them in metabolism phenotypes according to genotype meeting CPIC guidelines. Europeans represented most of the genotyped population (36%) and also had the greatest proportion of poor metabolizers (average, 5.4%), while Asians, Oceanians, and Middle Eastern populations, showed rates lower or equal to 1%. Ultra-rapid metabolizers were most represented in Oceanian (21.2%), Ashkenazi Jewish (11.5%), and Middle Eastern (11.2%) populations, with the lowest proportion in subjects from East Asia (1.4%). In spite of such efforts, the great genotypic variation in *CYP2D6* confers confounding effects to the actual metabolism phenotype observed in individuals, and clinical studies may not always reflect an accurate prediction of treatment response, tolerability, or even pharmacokinetic parameters. It is also important to note that most of these clinical studies are non-prospective, have great heterogeneity in design, often have low subject numbers and do not control for environmental or drug–drug interactions (Eum et al. 2016).

Fifteen antipsychotics are major or minor substrates of CYP2D6 including most options for BD treatment, with quetiapine (CYP3A4, CYP3A5) and ziprasidone (CYP1A2, CYP3A4) as notable exceptions (Eum et al. 2016). There is variability in the magnitude of change in 2D6 poor-metabolizers’ antipsychotic serum concentrations and/or half-life for primary CYP2D6 antipsychotic substrates [i.e., 1.7X increase in aripiprazole serum concentration (Hendset et al. 2007), 2X increase and 7X increase in half-life of aripiprazole and risperidone respectively (Eum et al. 2016)]. A recent large retrospective cohort of *CYP2D6* genotyped subjects treated with either risperidone (N = 1288) or aripiprazole (N = 1334) found statistically significant decreased metabolic ratios (i.e. metabolite/parent drug) for both drugs in poor and intermediate metabolizers; while greater metabolic ratios for ultra-rapid metabolizers, were only statistically significant for risperidone (Jukic et al. 2019). Moreover, poor and intermediate metabolizers showed increased risperidone and aripiprazole active moiety. These two drugs seem to present the greatest caution in 2D6 poor-metabolizer dosing; for further information on *CYP2D6* and dosing precautions see Table 1.

A meta-analysis found no association between *CYP2D6* genotyping and psychosis treatment efficacy in schizophrenia (Fleeman et al. 2011; Muller et al. 2012) however, it is important to point out that the authors found great variability of methodology and outcomes. The same study found, when including only prospective studies, a positive association between genotype, quantified only as mutant (i.e., not normal) vs wild type (i.e., normal), and tardive dyskinesia (Fleeman et al. 2011). In the retrospective study mentioned above, Jukic et al., found dose reductions from clinicians in both risperidone (19%) and aripiprazole (15%) poor metabolizers, however, only risperidone poor and ultra-rapid metabolizers show significantly higher switching to other antipsychotic agents; with no metabolizing phenotype showing greater switch to other agents in the aripiprazole group (Jukic et al. 2019). Performing prospective studies in BD will be of paramount importance before reaching more applicable clinical interpretations of *CYP2D6* genotyping and antipsychotic prescription.

Studies associating *CYP2D6* genotype and antidepressant pharmacokinetics and treatment response have been mostly performed in major depression. In patients treated with escitalopram (Ng et al. 2013; Hodgson et al. 2014, 2015), venlafaxine (Ng et al. 2013; Taranu et al. 2017) and nortriptyline (Hodgson et al. 2014, 2015), there was no association between metabolizing phenotype of CYP2D6 and response to these agents. The sole exception is the Lobello et al. (2010) study, which reviewed four placebo-controlled studies (n = 464) where 2D6 poor metabolizers, in comparison to extensive metabolizers, had significantly higher levels of serum parent compound venlafaxine, lower levels of active metabolite O-desmethylvenlafaxine, reduced baseline to endpoint change in depression scores, and significantly lower rates of treatment response and remission.

There is limited evidence that CYP2D6 poor-metabolizers were more likely to discontinue antidepressants (Berard et al. 2017). Antidepressant induced mania (AIM) has been reported in 3 bipolar depressed patients with a 2D6 poor metabolizer phenotype when prescribed 2D6 metabolized antidepressants (Sanchez-Iglesias et al. 2016). Similar to antipsychotics, *CYP2D6* genotyping does not have a clear role in clinical decision for antidepressant prescription in bipolar patients, further studies, especially regarding the AIM risk warrant further exploration.

### CYP2C19

Highly polymorphic-but significantly less than *CYP2D6*, *CYP2C19* is mainly involved in the metabolism of tricyclic and selective serotonin reuptake inhibitors (SSRI) antidepressants, and benzodiazepines. Its genotype status influences the concentrations of amitriptyline and imipramine, and precautions are suggested for poor metabolizers (Hicks et al. 2015, 2017). Citalopram, escitalopram and sertraline show higher serum concentrations in CYP2C19 poor metabolizers, however, in contrast with the above, the wide therapeutic window of these drugs suggest that their effects may be more difficult to simply categorize by their metabolic phenotype (Spina and de Leon 2015). Nonetheless, a recent study by Jukic et al. (Jukic et al. 2018) in more than two-thousand Europeans with multiple psychiatric diagnosis, genotyped for *CYP2C19* and medicated with escitalopram, showed that both poor and ultra-rapid *CYP2C19* metabolizers predicted greater switching from escitalopram to another antidepressant. In this same study, *CYP2C19* poor metabolizers showed greater odds of having lower than threshold therapeutic levels compared to extensive metabolizers; while this makes sense for ultra-rapid metabolizers, this is counterintuitive to poor metabolizer pharmacokinetics and may represent reduced treatment adherence (Jukic et al. 2018). In contrast, Fabbri and colleagues (Fabbri et al. 2018) found in a meta-analysis that *CYP2C19* poor-metabolizers had higher side-effects compared to extensive metabolizers at weeks 2–4, however, also showed higher symptom improvement and remission in major depression when treated with citalopram or escitalopram. A 10-year retrospective cohort found that *CYP2C19* poor metabolizers were more frequently observed in bipolar (9.8%) vs. unipolar subjects (0.6%, p = 0.003) (Veldic et al. 2019). The apparent impact of *CYP2C19* genotype in treatment response to citalopram and escitalopram in depression, together with the observations of differences in *CYP2C19* pharmacogenetics of bipolar depressed patients, warrants further exploration.

### CYP2C9

*CYP2C9* shows similar polymorphic variation to *CYP2C19*, but its role in the biotransformation of psychotropic drugs is minor (Spina and de Leon 2015). Of relevance, the secondary role it has in adult valproate metabolism seems to become clinically crucial in children, where it is responsible for most of this drug’s metabolism. Thus, its genotyping is recommended by some experts in this population (Monostory et al. 2019). CYP2C9 also modifies fluoxetine’s CYP2D6 metabolism but with an unknown clinical impact (Llerena et al. 2004).

Other pharmacokinetic genes such as *ABCB1* and *UGT1A4* are mentioned in Table 1.

Ethnic, environmental and drug–drug interaction variables also provide an enormous source of variability to the final metabolic phenotype expressed by the patient. Thus, bioinformatic tools based on systems biology will likely be needed in order to generate more intuitive models and clinical decision tools, based on pharmacokinetic PGT (McGraw et al. 2018).

## Targeted candidate genes in pharmacodynamic drug response/adverse event

Pharmacodynamic pharmacogenetic candidate gene studies of most bipolar drug classes have been performed, with genetic variants selected mechanistically from neurotransmission, gene transcription, neuroplasticity, intracellular messenger cascades, and other pathways. However, our drug mechanism of action understanding is incomplete and limits hypothesis-driven design of candidate gene studies and their interpretation; moreover, current PGT may potentiate the intrinsic risk for false positive discoveries (Farrell et al. 2015). Nonetheless, we discuss the findings from such candidate gene studies so that clinicians who are faced with PGT DST, understand how the genetic variants included in these platforms were selected and why they should be careful with their interpretation.

### Lithium

Lithium is the gold-standard mood stabilizing agent, with an extensive clinical evidence database for acute mania, bipolar depression, maintenance treatment (Bauer and Gitlin 2016), and suicidality prevention (Cipriani et al. 2013; Song et al. 2017). Clinical markers of response suggest that distinct patient groups may be more responsive to lithium (Grof et al. 1993; Post et al. 2016) suggesting a possible familial trait suggestive of genetic transmission (Grof et al. 1993, 2002, 2009). Lithium has been the most extensively studied of BD medications at the pharmacogenetic level and comprehensive reviews are available covering this topic (Alda 2015; Pisanu et al. 2016; Budde et al. 2017; Pickard 2017; Serretti 2017; Pisanu et al. 2018b).

Lithium mechanism of action involves multiple molecular mechanisms (Li et al. 2012). Thus, pharmacogenomic studies based on candidate genes have focused in genes implicated in many of these pathways. Neurotransmission genes for instance, largely based in monoamines—many of which are included in PGT—did not prompt any sufficiently robust associations of lithium treatment response (Budde et al. 2017; Pisanu et al. 2018b). Also included in PGT, the genetic single nucleotide polymorphism (SNP) rs6265, Val66Met, of *BDNF*, largely used to study neuroplasticity, showed mixed results (Michelon et al. 2006; Dmitrzak-Weglarz et al. 2008, Drago et al. 2010; Wang et al. 2012). GSK-3β is inhibited by lithium and, it is involved in neurogenesis, plasticity, and transcription through the Wnt canonical signaling pathway (Valvezan and Klein 2012). However, no robust evidence was found or replicated for *GSK3B* genetic variants (Michelon et al. 2006; Szczepankiewicz et al. 2006; Sathur Raghuraman et al. 2018); nor in other variants involved in genetic transcription, neuronal survival and plasticity, as SNPs in the CREB family (Mamdani et al. 2008) in association to lithium treatment response. Similarly, other variants related to inositol metabolism, involved in lithium’s mechanism of action, did not resulted in strong replicable results (Pisanu et al. 2016).

Lithium renal adverse events are an important though rare concern in BD treatment (Shine et al. 2015). To better inform lithium safety, a pharmacogenetic study of urinary concentration phenotypes were assessed in a group of 78 BD patients receiving lithium for a mean of 16 ± 9 years; in association with the *GSK3B*-50 C/T polymorphism, the authors found a statistically significant, though very modest association, not subjected to multiple testing correction, between the C-allele and kidney function, encouraging larger studies to better ascertain the potential role of *GSK3B* in informing lithium renal toxicity (Rybakowski et al. 2013).

### Valproic acid

Valproic acid (VPA) is recommended in the treatment of bipolar mania, depression and maintenance (Grunze et al. 2013, 2018; Yatham et al. 2018). Sodium channel blockade is one of VPA’s proposed mechanisms of action. Genetic variation in the *SCN* family genes, specifically SCN2A, which encodes of the sodium channel (Haug et al. 2001), has shown mixed evidence of association with VPA response (Haerian et al. 2013; Li et al. 2016); it is important to reference that these studies were conducted in epilepsy patients and anticonvulsant and mood stabilization therapeutic mechanisms of action may differ. Included in PGT, *CACNA1C* and other calcium channel coding genes have been tested for VPA efficacy, but they prompted negative results (Lv et al. 2015), again, these studies were conducted in epilepsy patients. In bipolar populations, VPA response showed a positive association with XBP1-116 C/G polymorphism, but replication is needed (Kim et al. 2009). *GNB3* variants have also been associated with metabolic abnormalities in cross-over (Chang et al. 2010) and prospective studies (Chen et al. 2017) of BD patients treated with VPA.

### Carbamazepine and lamotrigine

Although rare, serious dermatologic adverse events are observed with antiepileptic mood-stabilizers. Carriers of the HLA-B*15:02 allele in Asian population, specifically of Han Chinese descent, are at risk of developing severe hypersensitivity reactions in association with carbamazepine, lamotrigine, and phenytoin treatment (Bloch et al. 2014); moreover, the FDA recommends PGT in patients of Asian ancestry for this specific variation before initiating carbamazepine treatment (Drozda et al. 2018). The generalizability to other anticonvulsants or other racial ethnic groups in BD has not been investigated.

### Atypical antipsychotics

Weight-gain and other metabolic dysfunction is a major concern in BD treatment, especially regarding second-generation antipsychotics (SGAs) and mood-stabilizers. A recent meta-analysis of the genetic risk of anti-psychotics, predominantly SGAs-induced weight gain, explored 38 SNPS from 20 different genes in 6 independent samples (N = 6770) with predominant Caucasian and Asian ancestry (Zhang et al. 2016). 13 SNPs from 9 genes, namely *ADRA2A, ADRB3, BDNF, DRD2, GNB3, HTR2C, INSIG2, MC4R* and *SNAP25* showed statistically significant associations with antipsychotic-related weight gain (P-values < 0.05–0.001), while SNPs in *ADRA2A, DRD2, HTR2C,* and *MC4R* had the largest effect sizes (Hedges’ g’s = 0.30–0.80, ORs = 1.47–1.96 (Zhang et al. 2016). Important limitations to this meta-analysis include important heterogeneity, from selected antipsychotic agents, previous exposure to antipsychotics, time of exposure; also, the lack of multiple testing analysis. As with most genetic association studies, further functional analysis of these SNPs is needed before reaching a complete understanding of their actual biological impact on the mentioned genes or else. *ADR2A, BDNF,* and *DRD2* are included in most PGT commercial assays, however, the variants included in these tools are most of the time proprietary and thus it is unknown whether they test for the SNPs found in Zhang et al.’s study. Other efforts in understanding weight gain, specifically in BD, secondary to SGAs or mood stabilizers in 486 Systematic Treatment Enhancement Program for Bipolar Disorder subjects that did not identify significant candidate genes of weight gain liability (Creta et al. 2015).

### Antidepressants

Clinical recommendations have been developed to address antidepressant use in bipolar depression hopefully reducing “the striking incongruity” between the widespread use of antidepressants in BD and the limited evidence that supports their use (Pacchiarotti et al. 2013). One concern of antidepressant use in BD is risk of AIM. A meta-analysis of controlled trials of antidepressants in bipolar depression reported a 12.5% rate of treatment emergent mania (Tondo et al. 2010). While there are clinical factors identified with AIM (Frye et al. 2006, Goldberg et al. 2007), there is increasing investigation of genetic markers in this drug related adverse event. The most studied genetic association for AIM has involved the serotonin transporter gene (*SLC6A4*), that encodes the protein in charge of serotonin synaptic reuptake, and the variants involved in its genetic expression. There are predominantly 2 well-known polymorphisms: (5-HTTLPR) with long (L) and short (S) allele variants and a second intron variable number of tandem repeats (VNTR). 5-HTTLPR association to AIM has been studied with meta-analysis showing conflicting information (Daray et al. 2010; Biernacka et al. 2012). A more recent meta-analysis combining the Mayo Clinic Bipolar Biobank with 5 prior AIM studies, provided marginal evidence of association for the S-allele of 5-HTTLPR with AIM (*p* = 0.059). On the other hand, haplotype analysis including SNP rs25531 (A/G), and the intron 2 VNTR (9, 10, 12 repeat alleles) showed that the L-A-10 haplotype was associated with a reduced risk of AIM (*p* = 0.012) (Frye et al. 2015). *SLC6A4* variation is included in PGT commercial testing, however, before using it to inform AD prescription in BD, further exploration of the relationship between *SLC6A4* variation and risk of AIM is needed. For instance, intron 2 VNTR and other SNPs with an impact in *SLC6A4* expression, need to be employed in risk calculation, rather than focusing exclusively on the promoter long/short variant (Frye et al. 2015).

## GWAS of drug response/adverse event

GWAS employ an agnostic or untargeted approach and do not rely on mechanistic hypothesis. They have prompted the most promising results regarding genetic markers of treatment response in BD.

### Lithium

Most pharmacogenetic GWAS of BD have largely focused in Lithium. Initial efforts showed promising SNPs of risk, but they did not reach genome-wide significance of *p* ≤ 5 × 10^−8^ (Perlis et al. 2009; Squassina et al. 2011). The first genome-wide significant finding associated with lithium response, assessed by retrospective Alda scales, was achieved with a relatively small population (N = 294) of Han Chinese BD-I patients, and was replicated in an independent population (N = 100) (Chen et al. 2014). A robust association was observed for rs17026688 (p = 5.5 × 10^−37^) and rs17026651 (p = 2.52 × 10^−37^), variants in strong linkage disequilibrium (LD) in the *GADL1* gene, and replicated (p = 9.19 × 10^−15^ for each SNP). Moreover, they showed a 93% sensitivity in predicting lithium response (Chen et al. 2014). *GADL1* encodes a protein similar to GABA metabolism enzymes, suggesting biological plausibility of this finding. Furthermore, the effect-size of the association for the T-allele carriers and lithium response was enormous: 88.5 [95% confidence interval 41.4–198.0] (Chen et al. 2014). A study of this variant in a candidate gene study of Han Chinese patients additionally suggested a significant association with less recurrence and thus as a potential marker of lithium maintenance treatment (Chen et al. 2016). *GADL1* SNPs were not further replicated outside of this initial work (Hou et al. 2014; Ikeda et al. 2014; Cruceanu et al. 2015; Kotambail et al. 2015), however, Asian ancestry replications included both Han Chinese and Japanese populations (Hou et al. 2014) and Japanese-only populations (Ikeda et al. 2014). Moreover, the phenotype in the Chen et al., original study was narrower than further replications (Chen et al. 2014). An analysis of gene expression found no activity of *GADL1* in post mortem brain studies from individuals with BD (Birnbaum et al. 2014). The authors of this expression analysis hypothesized that, given the greater expression of *GADL1* in the kidney and its involvement in renal function (Liu et al. 2012), *GADL1* association to treatment response may be rather due to a renal function phenotype (Birnbaum et al. 2014).This is an important lesson in pharmacogenetics, showing the difficulty in interpreting and replicating even the most promising pharmacogenetic variants. Given its original and strong association with lithium response but further limitations in biological interpretation and replication, *GADL1* variants should be regarded with caution as potential pharmacogenetic markers of BD.

The largest pharmacogenetic consortium of lithium, the International Consortium on Lithium Genetics (ConLiGen), performed a GWAS in 2563 “bipolar spectrum” patients—mainly bipolar 1—from 22 participating sites, and showed a genome-wide significant association with a group of SNPs in a single region of chromosome 21 (Hou et al. 2016). This region has two genes that code non-coding RNAs, which in turn could be involved in gene expression (Hou et al. 2016), however, the actual functional effect of these variants is yet to be fully investigated. Interestingly, a prospective sample (N = 73) showed association with this region and lower relapse rates in 2-year follow-up (Hou et al. 2016). Replication, biological and clinical interpretation of this finding remains to be elucidated. Similar findings were found by the same group, showing an inverse relationship between lithium response and schizophrenia polygenic risk (Amare et al. 2018). Moreover, a cross-trait meta-GWAS found 15 genetic variants that may have overlapping effects on lithium treatment response and susceptibility to SCZ; bioinformatic analysis of these variants suggested the involvement of the HLA antigen complex and inflammatory cytokines (Amare et al. 2018). HLA members are included in some commercial PGT, however, their role in establishing lithium response is not yet validated. A GWAS performed in bipolar 1, 2, not specified and schizoaffective disorder, patients from Sweden and the United Kingdom with subjective (N = 2698) and objective (N = 1176) measures of treatment response, showed no genome wide significant results comparing lithium response between BD patients. However, when compared to healthy controls, a variant from imputation, prompted a validated genome-wide significant result in rs116323614 (*p* = 2.74 × 10^−8^), located in *SESTD1.* The later gene seems to be involved in phospholipid synthesis, which are potential lithium targets (Song et al. 2016). The biological and clinical relevance of this association remains to be determined. Not only should they be regarded with caution in PGT but, to our knowledge, none of these variants are available for PGT commercials assays to date.

### Clozapine

Clozapine is a SGA used in treatment resistant BD patients (Frye et al. 1998). In spite of its evident clinical benefit, clozapine use is limited due to clozapine-induced agranulocytosis/granulocytopenia (CIAG). Significant pharmacogenetic studied have explored this phenomenon, as recently reviewed by Numata et al. (2018). *S*everal GWAS have been conducted, an initial effort by Goldstein et al., on 161 CIAG cases and 1196 controls of European descent, found genome-wide associations for variants in *HLA*-*DQB1* and in *HLA*-*B* (Goldstein et al. 2014). In meta-analysis, only the HLA-DQB1 variant showed a nominally significant independent replication for a variant in HLA-DQB1 (OR = 15.6, P = 0.015, positive predictive value = 35.1), while a novel variant in rs149104283, an intronic transcript of *SLCO1B3* and *SLCO1B7*, was associated with CIAG (OR = 4.32, P = 1.79 × 10^−8^) (Legge et al. 2017), but was not replicated in a Japanese sample (Saito et al. 2017). A GWAS in Japanese samples including 50 cases of CIAG vs. 2905 controls, identified rs1800625 in the HLA region, particularly an association of HLA-B*59:01 with CIAG (Saito et al. 2016).

Dermatologic severe reactions to mood stabilizers and CIAG genetic risk, are for now better understood in Asian ancestries, thus limiting the use of PGT to this population.

No prospective trials have followed PGT and safety/tolerability phenotypes. Interestingly, a retrospective survey associating efficacy and tolerability in MD and BD patients with PGT, observed accordance between side effect tolerability phenotype and the 15 evaluated genes (70.6%), however, this latter association was not significant (p = 0.71) (Tonozzi et al. 2018).

## Potential role of DST that incorporate multiple genotype/phenotype drug recommendations

A number of commercially available platforms have been developed that rapidly assess pharmacokinetic and pharmacodynamic variation and develop a proprietary DST or algorithm to individualize treatment selection. A narrative review by Bousman and Hopwood (Bousman and Hopwood 2016) found 22 commercial companies offering PGT to five countries most prevalent in the USA (n = 13), followed by Australia and Canada (both n = 3). The majority of these platforms have focused on antidepressants in major depressive disorder primarily and antipsychotics in schizophrenia secondarily. Many of the pharmacogenetics candidate genes identified in response or adverse studies are commercially available as part of a DST. The extent of existing literature or clinical evidence base reviewed to assign a specific genotype a phenotypic pharmacokinetic (i.e., poor metabolizer) or pharmacodynamic (i.e., short form vs long form serotonin transporter) classification for each drug of the DST is proprietary. There is early work to suggest that different commercial products vary substantially, both in laboratory analysis of genotype and subsequent phenotypic classification (Bousman and Dunlop 2018).

There are early uncontrolled observations investigating the efficacy of PGT. Tonnozi et al. (2018) reported PGT of 15 genes (specific variants not reported) in 352 patients with major depression and BD. They reported that more than 60% of the self-reported retrospective treatment response and phenotype outcomes agreed (p = 0.001) and more than 70% of the reported side effect tolerability and phenotype outcome agreed, but this latter association was not significant. The retrospective nature of this association is a clear limitation. Prospective studies of DST have been compared to treatment as usual (TAU).

The Genomics Used to Improve Depression Decisions (GUIDED) trial is the largest study to date that randomized 1167 patients with major depression TAU vs genotype guided care. The primary outcome measure, baseline to endpoint in the Hamilton Depression Rating Scale, showed no significant difference between treatment groups, but secondary outcomes of treatment response and remission rates were statistically significant higher in in guided care vs TAU, though it is not clear if multiple testing was accounted for (Greden et al. 2019). Furthermore, patients randomized to guided care who switched from non-concordant treatment to concordant treatment, defined as agreement between DST recommendation and actual treatment selected, in comparison to those who stayed on non-concordant treatment, achieved a significant reduction in depressive symptoms and higher rates of response and remission. A recent meta-analysis of 5 studies, including Greden et al. (2019), reported that patients randomized to genotype guided care (n = 887) were 1.71 (95% CI 1.17–2.48, p = 0.005) times more likely to achieve remission than those patients randomized to TAU (Bousman et al. 2019). While the field continues to investigate these DST and attempt consensus as to their clinical utility, none of these studies have systematically investigated antidepressants or antipsychotics in BD. A small retrospective study of 30 BD-I and BD-II patients reported a similar observation to Greden et al. (2019) that as treatment concordance rates increased (i.e., genotype guided recommendation and actual treatment selected) from 13% at baseline and 40% at 3-month follow up, symptom severity as measured by the Clinical Global Impression—severity measure decreased (Ielmini et al. 2018). The bipolar pharamacopoiea would clearly advance where clinical treatment could be more individualized in reducing the risk:benefit ratio of antidepressants (i.e., AIM: depression efficacy) and atypical antipsychotics (tardive dyskinesia or cardiometabolics: depression efficacy).

There are two important examples of commissions dedicated to the evaluation of PGT in all medicine specialties. The Evaluation of Genomic Applications in Practice and Prevention (EGAPP) working group, part of the Centers of Disease Control and Prevention, developed a systematic process for evaluating genetic and PGT; they reviewed the utility of CYP450 PGT in major depression SSRI prescription more than 10 years ago and concluded, based on criteria of analytical validity, clinical validity, clinical usefulness, among other ethical/social aspects, that more research was needed before the benefit of PGT could be determined (E.o.G.A.i.P.a.P.E.W. Evaluation of Genomic Applications in Practice and Prevention (EGAPP) Working Group 2007). Concern was raised that important evidence from smaller pharmacogenetic trials and meta-analysis were not included that may have excluded important safety considerations related to CYP2D6 genotyping (Mrazek 2010). Commissioned by the National Health Service, the UK Genetic Testing Network (UKGTN) uses similar-to EGAPP but more extended criteria, to determine the benefit of PGT (UKGTN 2019b). Unfortunately, only dementia PGT is part of the psychiatry-related reports generated by this organization (UKGTN 2019a, b). However, using UKGTN modified criteria, Bousman and Hopwood (Bousman and Hopwood 2016) developed levels of evidence for pharmacogenetic testing variants available in commercial tests. Related to antidepressant therapy, a number of important development considerations were reviewed including: (1) 53% of the 46 tested genes had only preliminary or low supporting evidence (20% met criteria for the highest level), (2) only 60% of *CYP2D6* and *CYP2C19* metabolism-related variants were included in the commercial tests, (3) SNPs included per gene were not always reported in the commercial test, (4) limited availability of drug–drug and drug–gene interaction tools, and (5) not accounting for additional environmental effects and ancestry.

PGT also needs to be cost-effective and studies on willingness-to-pay show patient’s preference for PGT, in order to avoid trial-and-error prescriptions, medication changes and adverse events, is significant (Herbild et al. 2008, 2009). Early first investigations of PGT in mood and anxiety disorders appears promising. In a health claims dataset propensity-score, matched 6-month, case–control analysis, individuals with a mood/anxiety disorder (14.8% bipolar) who received PGT (n = 817), in comparison to similar mood/anxiety disorder individuals who did not receive PGT testing (n = 2745), recorded 40% fewer emergency room visits and 58% fewer inpatient all cause hospitalizations (Perlis et al. 2018). While overall 6-month number of psychotropic medications did not differ, overall costs were estimated to be nearly $2000 lower in the PGT group.

## Conclusions

DST based on the pharmacogenomics evidence base for BD are significantly underdeveloped in comparison to antidepressants for major depressive disorder and antipsychotics for schizophrenia. PGT commercial assays provide a few robust clinical applications for safety concerns raised by BD treatment, especially in cutaneous side effects of mood-stabilizers in Asian populations. Other potential uses in BD are mostly limited to metabolic phenotype associated variants. However, in comparison to genetic variation associated with adverse event or quantifiable biological process (i.e., rash, QTc prolongation), the bar to establish pharmacogenomic efficacy is significantly higher and to date, the evidence base for treatment recommendations is significantly less (Ahmed et al. 2018). It is important to note that most of these commercial assays vary in content—which may be not fully disclosed—of the genetic tests provided. Moreover, they may provide limited interpretation tools.

It is possible, but yet not investigated, that pharmacogenetic phenotyping in BD may differ by disease phase (e.g., mania, depression, maintenance), clinical components or subphenotypes (e.g., rapid cycling, obesity, binge eating), presence/absence and composition of multimodal drug therapy, environmental influences that magnify or mitigate genetic variation, and overall illness staging (at-risk, first episode, chronic illness with loss of euthymia and functional baseline).

The complexity of BD genetics and, the heterogeneity in BD drug-related phenotypes, are important considerations for the design and interpretation of BD PGT. As BD genetic risk is better understood by additional GWAS and functional genomic studies, the underlying neurobiology of the illness will provide better guidance for genomic testing of pharmacotherapy interventions. Furthermore, risk calculating tools would need to go beyond PGT, and include other important markers of risk, namely clinical risk factors, neuroimaging findings, among others; to better achieve precision medicine in BD (Perlis 2016).

The fast growing consortiums and GWAS studies on lithium response seem one of the most promising avenues for BD pharmacogenomics. Variants in *GADL1* and genes coding non-coding RNAs have been associated with this phenotype. However, these studies have thus far taught us important lessons, such as the one on Chen et al., *GADL1* study. In spite of showing genome-wide associations and large effect sizes, further investigation on *GADL1* did not support its association with lithium response. Moreover, functional studies seem to suggest that the *GADL1* SNPs associated with lithium response, do not have an effect on its genetic expression at the brain level. This is a remarkable reminder of the caution needed when interpreting pharmacogenetic studies, a precaution that is even more crucial before PGT reaches clinical implementation. It is also important to bear in mind that genetics are only a step in the complex “omic” levels that may explain the heritability of a given phenotype. Thus, PGT in BD need to be complemented with epigenetics, gene expression, proteomic, etc., and tested in cellular models such as the ones, for example, provided by induced pluripotent stem cells. Relevant examples of genomic tool integration in BD are emerging. Pisanu et al. (2018a) integrated GWAS lithium response findings in cellular lines, measured their effect on gene expression and, integrating these technologies, they found a potential target in a zinc-finger protein coding gene. Induced pluripotent stem cell lines have revealed hyperexcitability phenotypes in BD neurons that are reversed in lithium response patients (Mertens et al. 2015), underscored the importance of GSK3-β and other Wnt signaling members in the BD neuronal cell-lines (Madison et al. 2015), and elicited a promising new “pathway of lithium response”, that governs the phosphorylation of *CRMP2,* which seems to be indirectly modulating GSK3-β (Tobe et al. 2017). The implementation of in silico and machine-learning technologies, will also be crucial in integrating these findings to design intuitive models that help us mimic the biological complexity of genetic–phenotypic interactions.

The clinical applicability of PGT in psychiatry is in its infancy (Stern et al. 2018) and is far from reaching the robust impact it has for instance, in other medical disciplines such as oncology. Nonetheless, promising findings in pharmacogenomic markers of efficacy are discovered with increasing frequency, especially for lithium. Over the last 10 years, pharmacogenetics and other pharmacogenomic technologies have evolved significantly, promising results of remarkable relevance in neuroscience, pharmacology and biology. Their ultimate goal of generating precision medicine in BD, may be a reality not so far in our future.

## Data Availability

All data used in the review has been published and/or referenced.

## Abbreviations

BD: Bipolar disorder
DSM-5: Diagnostic and Statistical Manual of Mental Disorders
FDA: Food and Drug Administration
CPIC: Clinical Pharmacogenomics Implementation Consortium
PGRN: Pharmacogenomics Research Network
PGT: Pharmacogenomic testing
CYP: Cytochrome P450
VPA: Valproic acid
SGA: Second-generation antipsychotics
AIM: Antidepressant induced mania
SSRI: Selective serotonin reuptake inhibitors
SNP: Single nucleotide polymorphism
*SLC6A4*: Serotonin transporter gene
GWAS: Genome wide association studies
ConLiGen: Consortium on Lithium Genetics
CIAG: Clozapine-induced agranulocytosis
DST: Decision Support Tool
TAU: Treatment as usual
GUIDED: The Genomics Used to Improve Depression Decisions
EGAPP: The Evaluation of Genomic Applications in Practice and Prevention
UKGTN: UK Genetic Testing Network
